# Harnessing the power of structure‐based design: A new lease on life for cardiovascular drug development with apelin receptor modulators

**DOI:** 10.1002/ctm2.70116

**Published:** 2024-12-08

**Authors:** Junxia Zhang, Erdan Dong, Yan Zhang, Yan Zhang

**Affiliations:** ^1^ Department of Cardiology and Institute of Vascular Medicine, Peking University Third Hospital，Institute of Cardiovascular Sciences, School of Basic Medical Sciences，Peking University Health Science Center，State Key Laboratory of Vascular Homeostasis and Remodeling Peking University Beijing China; ^2^ Research Unit of Medical Science Research Management/Basic and Clinical Research of Metabolic Cardiovascular Diseases, Chinese Academy of Medical Sciences Haihe Laboratory of Cell Ecosystem Beijing China; ^3^ Research Center for Cardiopulmonary Rehabilitation, University of Health and Rehabilitation Sciences Qingdao Hospital (Qingdao Municipal Hospital), School of Health and Life Sciences University of Health and Rehabilitation Sciences Qingdao China; ^4^ Department of Pharmacology and Department of Pathology of Sir Run Run Shaw Hospital & Liangzhu Laboratory Zhejiang University School of Medicine Hangzhou China; ^5^ MOE Frontier Science Center for Brain Research and Brain‐Machine Integration Zhejiang University School of Medicine Hangzhou China; ^6^ Institute of Cardiovascular Diseases The First Affiliated Hospital of Dalian Medical University Dalian China

## 1 DRUG DEVELOPMENT TARGETING G PROTEIN‐COUPLED RECEPTORS

G protein‐coupled receptors (GPCRs) are crucial cell surface receptors involved in many physiological processes.^1^ Around 30%–40% of existing drugs target GPCRs, leading to treatments for cardiovascular, metabolic and neurological diseases.^2^, ^3^ Recently, biased agonists, which selectively activate specific GPCR pathways while avoiding others, have garnered attention for their potential to reduce side effects and enhance therapeutic efficacy.^4^ A case in point is PZM21, a biased agonist of the μ‐opioid receptor (μOR), which preferentially engages G protein coupling over β‐arrestin, reducing traditional opioid‐related side effects such as addiction and respiratory depression.^3^ Researchers have elucidated distinct receptor activation states through atomic‐level simulations, proposing a “G protein‐first” activation model.^5^ The advent of high‐resolution GPCR structural biology, facilitated by techniques like cryo‐electron microscopy, is propelling the evolution of precision drugs tailored to these receptors.

## 2 APJ‐BASED DRUG DEVELOPMENT OF CARDIOVASCULAR DISEASE

Cardiovascular diseases remain the preeminent cause of mortality worldwide, with a marked escalation in incidence rates across all income levels, disproportionately affecting low‐ and middle‐income nations.^6^ Despite the stark need, only a minuscule fraction targeted cardiovascular diseases compared to oncological agents.^7^ This disparity underscores an imperative for more efficacious drug discovery endeavours within cardiovascular medicine.

The apelin receptor (APLNR) is a key regulator of cardiovascular function, influencing heart contractility, stroke volume and blood pressure.^8^ It is implicated in various cardiovascular diseases, including heart failure and pulmonary arterial hypertension.^9^ Apelin, an endogenous ligand of APLNR, functions as a balanced agonist activating both G‐protein and β‐arrestin pathways of APLNR. It has been proposed that the apelin‐induced cardioprotective effect is primarily attributed to Gi signalling of APLNR, whereas the β‐arrestin pathway of APLNR causes detrimental cardiac hypertrophy.^10^ The development of G‐protein‐biased APLNR agonists for heart failure has advanced significantly, with some partial agonists demonstrating enhanced cardiovascular benefits in both animal models and humans.^11^, ^12^ However, the problem of β‐arrestin signalling remains, which can limit the therapeutic potential of these medications. One notable example is MM07, a peptide designed to improve G protein signalling by establishing a disulfide bond via cysteine alterations.^13^ Another example, CMF‐019, is a small‐molecule APLNR agonist that selectively activates G protein pathways.^12^ Although G‐protein‐biased agonists are designed to decrease β‐arrestin activity, both MM07 and CMF‐019 engage β‐arrestin to some extent, albeit at lower levels than traditional agonists. This residual β‐arrestin activation poses safety risks since it is associated with different physiological and pathological processes to some degree raising valid safety concerns. Further optimization of these molecules, as well as a deeper understanding of the complex interplay between G protein and β‐arrestin signalling, will be crucial in the development of safer and more effective drugs.

**FIGURE 1:**
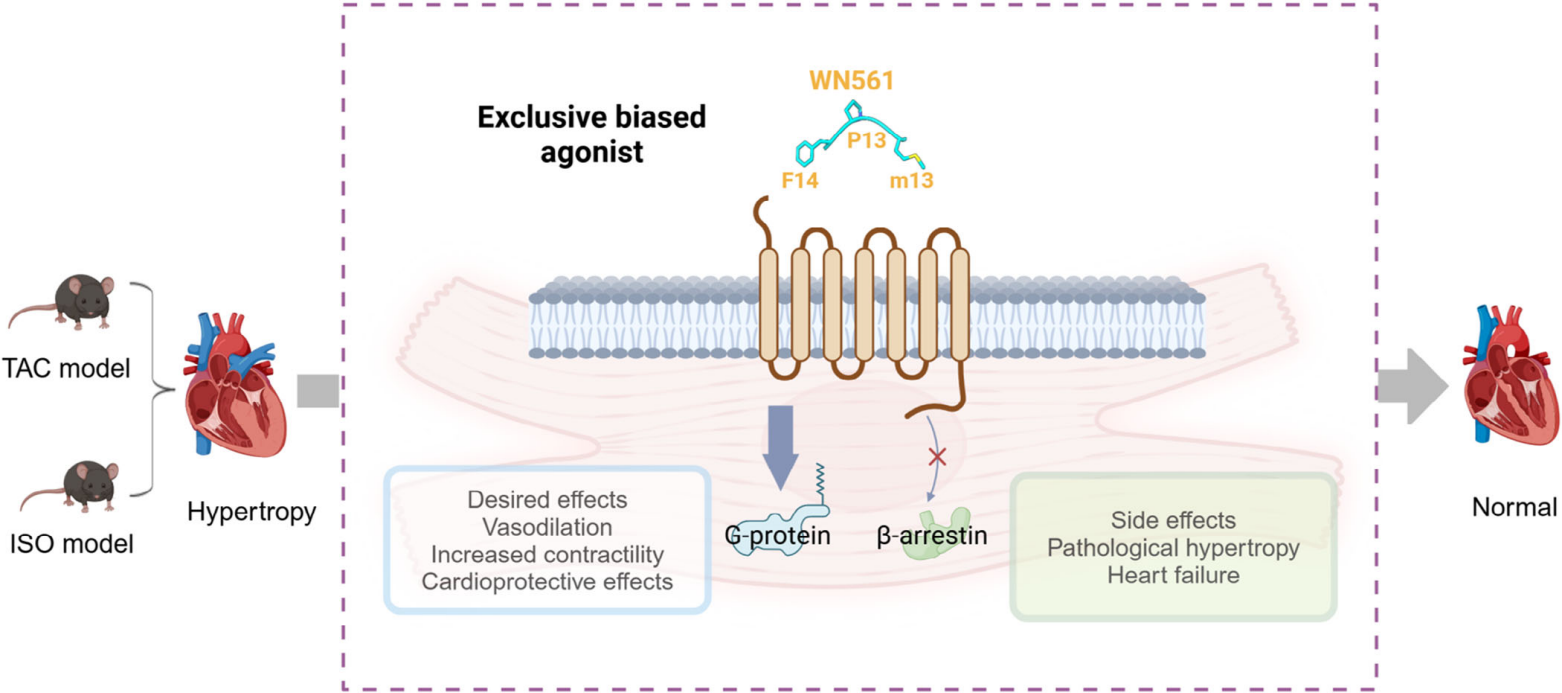
Mechanism of WN561, an exclusive G protein‐biased agonist, improving the pathophysiology of cardiac hypertrophy. WN561 selectively promotes the G protein signalling pathway downstream of the apelin receptor (APLNR), effectively bypassing the β‐arrestin‐mediated pathway. This selective activation leads to the alleviation of myocardial hypertrophy in mice, both with and without pathological stimuli.

Wang et al. are actively investigating new G‐protein‐biased agonists, such as WN561, which have shown promise in reducing cardiac hypertrophy and protecting cardiovascular function (Figure 1).^14^ The analysis of APLNR‐Gi1 complexes revealed “twin hotspots” in APLNR that are crucial for signaling bias, leading to the design of new G‐protein‐biased agonists like WN353 and WN561. These new compounds maintain G protein activity while minimizing β‐arrestin activation, offering promising avenues for therapeutic development. In studies, both apelin and WN561 treatment alleviated cardiac hypertrophy. However, apelin posed a risk of promoting hypertrophy in normal cardiomyocytes and mice, whereas the G‐protein‐biased peptide WN561 showed reduced potential adverse effects. We created a mouse model of cardiac hypertrophy which reflects a scenario where individuals develop cardiac hypertrophy due to harmful triggers that are later treated with medication or surgery. In clinical practice, diseases such as hypertension and valvular disease frequently result in pathological cardiac hypertrophy, a major risk factor for heart failure. Our findings indicate that in mice without isoprenaline pumps, previous G‐protein‐biased agonists (MM07 and CMF‐019) did not reverse cardiac hypertrophy but worsened it instead. In contrast, our newly developed agonist, WN561, alleviated myocardial hypertrophy in both the presence and absence of pathological stimuli (Figure 1). These findings suggest that WN561 may be a more suitable treatment option for patients with heart hypertrophy, even after the underlying triggers have been addressed.

## 3 FUTURE RESEARCH DIRECTIONS ON CARDIOVASCULAR DISEASE

Future research will prioritize the development of novel agonists like WN561, aiming to reduce side effects while effectively treating cardiovascular diseases. Clinical trials will be crucial to validate the safety and efficacy of Apelin/APJ system agonists. Additionally, exploring why APLNR‐biased agonists have different therapeutic effects still requires in‐depth investigation into the specific molecular mechanisms.

The APLNR holds significant promise as a therapeutic target for cardiovascular diseases, with the development of novel agonists like WN561 marking a pivotal step forward. These G‐protein‐biased agonists are designed to harness the beneficial effects on heart function while circumventing the adverse β‐arrestin signalling associated with traditional agonists. The future of cardiovascular drug development, much like the advancements in cancer classification and precision medicine, necessitates a departure from traditional, organ‐centric classification methods.^15^ The challenges of high costs, extended development timelines and the low success rate of translating discoveries into clinical applications have dampened industry enthusiasm for cardiovascular research. However, there is a pressing need, and indeed an opportunity, to enhance patient phenotyping and to adopt a more nuanced, molecular‐based approach to disease classification. This will lead to the establishment of platforms and methodologies that are not only innovative but also reflective of the complex biological mechanisms at play, thus broadening the horizon of therapeutic development for cardiovascular diseases.

## 4 CONCLUSION

In conclusion, by prioritizing the development of novel agonists such as WN561, future research aims to enhance treatment efficacy while minimizing side effects. The validation of these treatments' safety and efficacy will rely heavily on rigorous clinical trials. Simultaneously, a deeper exploration of the Apelin/APJ system's molecular mechanisms in various cardiovascular conditions is essential for advancing treatment strategies. The adoption of precision medicine in cardiovascular drug development is essential due to the limitations of traditional classification methods. It is leading the way to a new era of therapeutic development that is tailored to the intricate biological landscape of cardiovascular diseases.

## AUTHOR CONTRIBUTIONS

Yan Zhang (PKU) and Junxia Zhang conceptualized and wrote the commentary. Yan Zhang (PKU), Yan Zhang (ZJU), Erdan Dong and Junxia Zhang provided the funding.

## CONFLICT OF INTEREST STATEMENT

The authors declare no conflict of interest.

## ETHICS STATEMENT

Not Applicable
